# Possible molecular mechanisms linking air pollution and asthma in children

**DOI:** 10.1186/1471-2466-14-31

**Published:** 2014-03-01

**Authors:** Susanna Esposito, Rossana Tenconi, Mara Lelii, Valentina Preti, Erica Nazzari, Silvia Consolo, Maria Francesca Patria

**Affiliations:** 1 Pediatric Highly Intensive Care Unit, Department of Pathophysiology and Transplantation, University of Milan, Fondazione IRCCS Ca’ Granda Ospedale Maggiore Policlinico, Via Commenda 9, 20122 Milano, Italy

**Keywords:** Air pollution, Asthma, Lung disease, Particulate matter, Pediatric pulmonology, Respiratory tract infection

## Abstract

**Background:**

Air pollution has many effects on the health of both adults and children, but children’s vulnerability is unique. The aim of this review is to discuss the possible molecular mechanisms linking air pollution and asthma in children, also taking into account their genetic and epigenetic characteristics.

**Results:**

Air pollutants appear able to induce airway inflammation and increase asthma morbidity in children. A better definition of mechanisms related to pollution-induced airway inflammation in asthmatic children is needed in order to find new clinical and therapeutic strategies for preventing the exacerbation of asthma. Moreover, reducing pollution-induced oxidative stress and consequent lung injury could decrease children’s susceptibility to air pollution. This would be extremely useful not only for the asthmatic children who seem to have a genetic susceptibility to oxidative stress, but also for the healthy population. In addition, epigenetics seems to have a role in the lung damage induced by air pollution. Finally, a number of epidemiological studies have demonstrated that exposure to common air pollutants plays a role in the susceptibility to, and severity of respiratory infections.

**Conclusions:**

Air pollution has many negative effects on pediatric health and it is recognised as a serious health hazard. There seems to be an association of air pollution with an increased risk of asthma exacerbations and acute respiratory infections. However, further studies are needed in order to clarify the specific mechanism of action of different air pollutants, identify genetic polymorphisms that modify airway responses to pollution, and investigate the effectiveness of new preventive and/or therapeutic approaches for subjects with low antioxidant enzyme levels. Moreover, as that epigenetic changes are inheritable during cell division and may be transmitted to subsequent generations, it is very important to clarify the role of epigenetics in the relationship between air pollution and lung disease in asthmatic and healthy children.

## Background

Outdoor air pollutants can come from many sources and include both gaseous and particulate pollution. Air pollution arises in two ways: as primary pollutants emitted directly out of exhaust pipes and stacks and as secondary pollutants formed from the primary pollutants in the atmosphere in the copresence of sunlight, moisture or both [1]. The dominant anthropogenic origin of all these pollutants is the combustion of fossil fuels. In urban and suburban areas, transportation-related emissions are a major source of air pollution. The main transportation-related air pollutants include carbon monoxide, nitrogen dioxide (NO_2_), and particulate matter (PM), a complex mixture of chemicals and particles of which diesel exhaust particles (DEP) are the largest single source [2]. These compounds can arise from the pumping of gasoline, exhaust from the combustion of fuel, and the resuspension of settled road dust particles by moving vehicles. Other sources can include large industrial facilities, smaller industrial operations, nonpoint sources and natural sources [1].

Air pollutants have many effects on the health of both adults and children, but children’s vulnerability is unique. First of all, as a child’s lungs are still growing, early exposure to environmental pollutants can more easily alter lung development and lung function. Various studies have demonstrated an association between reduced lung growth and PM concentrations, and shown that air pollution alters lung development [3-6]. Gauderman *et al*. found that children with asthma living in an area with high PM concentrations showed reduced lung growth [6], and a very recent study of a birth cohort of 1,185 children has shown that long-term exposure to NO_2_ and PM with a diameter of ≤10 μm (PM_10_) was associated with a small but significant reduction in lung volume [7]. In a study of 3,168 Chinese schoolchildren, it was found that PM_10_ was primarily responsible for lung function impairment [8]. In another study linking modelled exposure to traffic-derived PM in the first years of life to lung function at the age of eight years, Schultz *et al*. observed that only exposure in the first year of life is associated with a significant reduction in forced expiratory volume in one second (FEV_1_) [9].

Secondly, children spend a lot of time outdoors (especially at times when PM concentrations are higher) and engage in physical activities that increase their breath rate, which leads to larger deposits of environmental pollutants in the respiratory tract. This is supported by the fact that the correlation between exacerbations of asthma and environmental pollution is closer in the summer, and the risk is reduced in the winter [10]*.*

Thirdly, young children are predominantly oral breathers, which means that the nasal filter is by-passed and more polluted particles can enter the lower airways [11].

Table 1 summarizes the population-based studies of the effects of air pollution in childhood [12-15]. In a prospective birth cohort study of 4,089 Swedish children, Nordling *et al.* found that exposure to traffic-induced air pollution during the first year of life was associated with an excess risk of persistent wheezing and sensitisation to inhaled allergens at the age of four years [12]. Similarly, Brauer *et al*. found a positive association between air pollution exposure, sensitisation to food allergens and respiratory symptoms at the age of four years in a birth cohort study involving 4,000 children in The Netherlands [13]. However, data from the long-term follow up of the PIAMA cohort suggest that the involvement of traffic-related air pollution in the onset of asthma by eight years of age may be limited to non-atopic asthma because the association between allergic sensitisation and air pollution was not significant [14]*.* The BAMSE Swedish birth cohort of 4,089 children followed up until they were 12 years old also documented a close association between air pollution and non-allergic asthma [15].

**Table 1:** Population-based studies of the effects of air pollution in childhood

**Authors**	**Year**	**Methods**	**Main conclusion**
Nordling *et al*. [12]	2007	The spatial distribution of nitrogen oxides from traffic (traffic-NOx) and inhalable particulate matter from traffic (traffic-PM10) in the study area was assessed with emission databases and dispersion modeling. Estimated levels were used to assign first-year exposure levels for children in a prospective birth cohort (n = 4089), by linking to geocoded home addresses. Parents in 4 Swedish municipalities provided questionnaire data on symptoms and exposures when the children were 2 months and 1, 2, and 4-year-old. At 4 years, 73% of the children underwent clinical examination including peak expiratory flow and specific IgE measurements.	Exposure to traffic pollution in early childhood is associated with an excess risk of persistent wheezing and sensitisation to inhaled allergens at the age of four years.
Brauer *et al*. [13]	2008	The development of asthmatic/allergic symptoms and respiratory infections during the first 4 yrs of life was assessed in a birth cohort study (n = approximately 4,000). Outdoor concentrations of traffic-related air pollutants were assigned to birthplace home addresses with a land-use regression model. They were linked by logistic regression to questionnaire data on doctor-diagnosed asthma, bronchitis, influenza and eczema and to self-reported wheeze, dry night-time cough, ear/nose/throat infections and skin rash.	Air pollution exposure in early childhood is associated with sensitisation to food allergens and respiratory symptoms at the age of four years.
Gehring *et al*. [14]	2009	Annual questionnaire reports of asthma, hay fever, and related symptoms during the first 8 years of life were analyzed for 3,863 children. At age 8, measurements of allergic sensitization and bronchial hyperresponsiveness were performed for subpopulations (n = 1,700 and 936, respectively). Individual exposures to NO_2_, PM2.5, and soot at the birth address were estimated by land-use regression models. Associations between exposure to traffic-related air pollution and repeated measures of health outcomes were assessed by repeated-measures logistic regression analysis.	Exposure to traffic-related air pollution seems to cause asthma in children monitored from birth to eight years of age.
Gruzieva *et al*. [15]	2013	The Swedish birth cohort BAMSE (Children, Allergy, Milieu, Stockholm, Epidemiological Survey) includes 4,089 children who were followed up with repeated questionnaires and blood samples for up to 12 years of age. Residential, daycare, and school addresses, time-activity patterns, emission databases, and dispersion models were used to estimate individual exposure to PM10 and NOx from traffic.	There is a moderate positive association between exposure to traffic-related air pollution in infancy and asthma in children aged up to 12 years, which is possibly stronger in the case of non-allergic asthma.

NO: nitrogen oxide; PM: particulate matter.

A number of epidemiological studies have raised questions as to how exposure to common air pollutants plays a role in the susceptibility to, and severity of respiratory infections. Ezzati *et al.* followed 93 infants and children in a rural zone of central Kenya for more than two years [16], and found that there was a significant association between the concentration of PM and the risk of developing an acute lower respiratory infection. Barnett *et al*. examined the effect of air pollution on daily hospital admission counts among children in Australia and New Zealand and found a statistically significant relationship, with the closest association between air pollution and asthma admissions of children aged 5–14 years [17]. Migliore *et al*. confirmed the close association between vehicular traffic and cough or phlegm in 33,632 Italian children and adolescents [18] and similar data have been confirmed by Hoek *et al*. in >45,000 children from 12 countries [19]. Moreover, it has also been observed that prenatal exposure to PM also increases susceptibility to recurrent broncho-pulmonary infections in early childhood in a dose–response manner [20].

In addition, although the interaction is not completely clear, it has recently been suggested that genetics and epigenetics play a role in the damage to airways caused by air pollution, and some therapeutic approaches based on this hypothesis have been proposed [21,22].

The aim of this review is to discuss the possible molecular mechanisms linking air pollution and lung diseases in children, also taking into account their genetic and epigenetic characteristics. PubMed was used to search for all of the studies published over the last 15 years using the key words: “air pollution” or “carbon monoxide” or “particulate matter” or “diesel exhaust particles” and “children” or “paediatrics”. Only articles published in English were included in the evaluation.

## Review

### Pollution-induced airway inflammation

One important mechanism by means of which air pollution causes lung injury is certainly the induction of a persistent inflammatory state mediated by the immune system [23]. Biomarkers such as 8-isoprostane and cytokines measured in exhaled breath condensate have been considered indicators of airway inflammation, and some clinical studies have shown that short-term exposure to traffic-related air pollutants can increase airway inflammation and/or oxidative stress in pediatric age [24-26]. In a recent pediatric study, Patel *et al*. found that a short-term increase in ambient black carbon from DEP and NO_2_ from vehicle emissions was associated with a decrease in the pH of the exhaled breath condensate of 36 adolescents (thus indicating increased airway inflammation and oxidative stress), with no difference between asthmatic and non-asthmatic subjects [24]. Another study of 224 asthmatic children found that those living closer to a major roadway had increased generalised airway and systemic inflammation as indicated by the lower pH of their breath condensate and their higher plasma concentrations of epidermal growth factor, which is associated with airway tissue remodelling in children [25]. Finally, short-term exposure to particulate matter (including PM_10_) is associated with higher exhaled breath concentrations of nitric oxide in childhood [26].

The molecular pathways through which airway inflammation induces lung injury have not been fully elucidated but numerous studies have demonstrated that they certainly involve increased IgE- mediated sensitisation to airborne allergens [27-35] and toll-like receptor(TLR)-mediated innate immune responses [36-42].

Regulatory T cells (T reg) seem to play an essential role in inhibiting the proximal pathways of allergic sensitisation and IgE production in response to allergen exposure [31], and Nadeau *et al.* made a pivotal step forward in our understanding of how air pollution influences IgE-mediated responses in asthmatic children by impairing T reg function [32]. Comparing groups of asthmatic and non- asthmatic children exposed to different air pollutant levels, they found that the children who lived in a more polluted place often showed hypermethylation of the transcriptional factor Foxp3, which impairs T reg cells and increases asthma morbidity. Like those of other recent studies, the findings of this study support the hypothesis that epigenetic mechanisms may form a link between genetic and environmental factors in the pathogenesis of asthma [33-35].

In relation to TLR-mediated innate immune responses in pollution-induced lung injury in asthmatic children, it is known that members of the TLR family help to defend against a variety of antigens and that they are signal transducers for exposure to pathogen-associated molecular patterns (PAMPs) such as lipopolysaccharide (LPS) and various mediators of inflammation released in response to tissue damage (i.e. damage-associated molecular pattern molecules or DAMPs) [36]*.* LPS is an endotoxin found on the cell membrane of Gram negative bacteria and one of the constituents of PM. Table 2 summarises the findings of the experimental studies concerning the interaction between TLRs and air pollution. Two studies have shown that TLRs 2 and 4 on human alveolar macrophages, and TLR2 on bronchial epithelial cells, are activated by bacterial components such as LPS attached to PM [37,38]. Genetic polymorphisms in TLRs may be the link between PM, passive smoking, NO_2_ and childhood asthma. *In vitro* and *in vivo* studies have demonstrated that ozone and LPS increase airway neutrophil counts, and that the patients’ responses to each were correlated, thus suggesting the presence of a common signalling pathway [39]. It has also been observed that mice exposed to ozone and LPS develop asthma as a result of the activation of TLR4 on the surface of inflammatory cells [40]. Similarly, TLR2 (−/−) and TLR 4 (−/−) mice show less ozone-induced airway hyper-responsiveness and neutrophilia than wild-type mice [41]*.* The role of TLRs in air pollution-induced immune responses has been confirmed by an epidemiological study of 916 children belonging to the PIAMA birth cohort study [42], which found that only the children with specific polymorphisms in the *TLR2* and *TLR4* genes were susceptible to the adverse effects of air pollution on asthma in a dose-dependent manner.

**Table 2:** Experimental studies of the interactions between toll-like receptors (TLRs) and air pollution

**Authors**	**Year**	**Methods**	**Main conclusion**
Becker *et al*. [37]	2002	Stimulation of interleukin-8 release from normal human airway epithelial cells with coarse PM2.5-10, fine PM2.5, and ultrafine particle fractions has shown that the coarse particle fraction has the greatest effect on the epithelial cells as well as alveolar macrophages. Since this fraction concentrates fugitive dusts and particle-associated microbial matter, it was hypothesized that epithelial cells may recognize PM through microbial pattern recognition receptors TLR2 and TLR4, as has been previously shown with alveolar macrophages.	Microbial components and TLRs are important players in alveolar macrophage-dependent inflammatory responses to PM.
Becker *et al*. [38]	2004	The hypothesis behind the present study was that not only particle-bound LPS, but also Gram-negative and Gram-positive bacteria are responsible for PM-induced stimulation of alveolar macrophage, and therefore that PM are likely to activate receptors involved in recognition of microbes.	Both airway epithelial cells and macrophages involve TLRs in recognising the molecules present in PM; epithelial cells use TLR2 and TLR4.
Hollingsworth *et al*. [40]	2007	Because ozone also alters clearance of pulmonary bacterial pathogens, the authors hypothesized that inhalation of ozone modifies innate immunity in the lung. To address our hypothesis, they exposed C57BL/6 J mice to either free air or ozone, and then subsequently challenged with an aerosol of *Escherichia coli* LPS.	Ozone exposure increases the pulmonary and systemic biological responses to inhaled LPS by priming the innate immune system.
Williams *et al*. [41]	2007	The authors investigated the sensing of ozone by TLR2, TLR4, and MyD88. Moreover, they evaluated the expression of inflammatory cytokines and TLR2, TLR4, and MyD88 in these mice.	TLR2- and TLR4-mediated inflammation induced by oxidative stress and the adapter protein MyD88, but not by TLR2 or TLR4, is important in mediating ozone-induced neutrophilia.

LPS: lipopolysaccharide; PM: particulate matter; TLR: toll-like receptor.

It has also been shown that the neutrophils attracted into the airways after exposure to ozone and endotoxin produce reactive oxygen species (ROS) that induce epithelial cell inflammation, airway hyper-reactivity and lung injury [39] by means of a complex mechanism that is not fully understood. Free radicals can directly induce the production and activation of pro-inflammatory mediators or indirectly induce the release of DAMPs after tissue injury. One clear example of this second pathway comes from studies of hyaluronan [43], a widely distributed anionic, non-sulfated glycosaminoglycan that is found in the epithelial extracellular matrix. Its fragmentation into low-molecular-mass forms can be the result of ROS release during tissue injury, and it has been demonstrated that these fragments become endogenous ligands for TLR4 and can activate the cytokine production of the innate immune response [43]*.*

Another inflammatory gene associated with airway inflammation is tumour necrosis factor (TNF), which is thought to affect the expression of pro-inflammatory cytokines. TNF polymorphisms seem to influence the functional response of the lungs to ozone, and the ozone-dependent risk of developing asthma [43].

All of the studies described above show that air pollutants induce persistent airway inflammation and increase asthma morbidity in children. The better definition of mechanisms related to pollution-induced airway inflammation in asthmatic children may make it possible to find new clinical and therapeutic strategies for preventing the exacerbation of asthma.

### Pollution-induced oxidative stress

Asthma is the pediatric respiratory disease that has proved to be most closely correlated with pollution-induced oxidative stress [44]. Pollutants can cause lung damage due to oxidative stress by acting directly on the production of free ROS, or indirectly by inducing inflammation. ROS are produced as a normal product of cell metabolism and induce cell damage by reacting with intracellular constituents such as DNA and membrane lipids [44]*.* Antioxidant redox systems and antioxidant enzymes neutralize ROS, but oxidative stress may induce posttranslational modifications in proteins modulating ROS activities. Emerging evidence suggests that environmental pollutants may promote allergic sensitisation: a recent pediatric study found that the risk of allergic sensitisation was increased by exposure to non-volatile polycyclic aromatic hydrocarbons (PAH) in children without the common *GSTM1* gene polymorphism, who seemed to be more susceptible to sensitisation by combined cockroach allergen and PAH exposure [45]*.*

Antioxidants such as glutathione help to reduce the epithelial cell inflammation and tissue injury generated by ROS. Adults with *GSTM1* null genotypes show reduced glutathione-S-transferase (GST) enzyme activity and consequently have higher sputum neutrophil and macrophage counts following ozone exposure, thus suggesting that polymorphisms in antioxidant enzyme genes may play a role in increasing pollution airway inflammation [46]*.* Other studies have demonstrated that children with the *GSTM1* null or *GSTP1* variant genotype are at increased risk of developing asthma when exposed to ozone or tobacco smoke [47,48], and a prospective study of 2,106 American children from 12 Southern California cities found that sequence variations in the genes of the glutathione synthesis pathway are associated with differences in susceptibility to the adverse effects of pollutants on developing lung function [49].

However, other evidence partially refutes the relationship between air pollution and polymorphisms in oxidative stress genes. The authors of a recent systematic review of 15 studies of antioxidant gene-pollution interactions (12 of which supported the presence of interactions) found only one study showing an interaction with the *GSTM1* null genotype, although five studies found interactions when *GSTM1* was evaluated together with other genes (mainly *NQO1*) [50]. Some evidence was found of an interaction with *GSTP1*, although with conflicting results in terms of the risk allele, whereas the results *GSTT1* were negative [50]. This means that it is not yet known which pollutants and which genes interact with each other. Although this systematic review included studies performed in asthmatic adults, its results maintain unanswered questions also for the pediatric age.

Other antioxidant enzymes that may be relevant to the impact of pollution on airway disease are catalase and myeloperoxidase. A study of 1,935 Californian students found that genetic polymorphisms in these two enzymes were jointly associated with acute respiratory illness as measured on the basis of respiratory-related school absences [51].

An attractive approach to reducing the effect of air pollutants such as DEP involves the induction of enzymatic antioxidant defences, especially in the case of subjects with at-risk genetic variants of key antioxidant enzymes [52]. Previous studies have found that antioxidant substances such as ascorbate (vitamin C) may protect the airways from the oxidative injury induced by exposure to air pollutants. Romieu *et al.* recruited 158 asthmatic children exposed to high levels of ozone in Mexico City in a double-blind study, and studied their pulmonary function in relation to their *GSTM1* polymorphism and dietary supplements of the antioxidant vitamins C and E [53]. It was found that the children with a *GSTM1* null genotype showed a greater decline in lung function due to ozone exposure and received greater benefit from antioxidant supplementation. Further studies are needed to confirm whether antioxidant supplementation is biologically meaningful in asthmatic children. As the public health goal of reducing children’s exposure to air pollutants is very difficult to achieve, the approach to reducing pollution-induced oxidative stress and consequent lung injury with the administration of antioxidant substances [52,53] could be interesting. However, further evidence is needed before administering antioxidant supplementation to children with asthma who seem to have a genetic susceptibility to oxidative stress, and further data should clarify whether this strategy would be useful also for the healthy population.

### Epigenetics

The word epigenetics is used to describe inheritable changes in gene expression due to non-coding changes in DNA [54]. Epigenetic modifications can alter the structure of DNA itself (such as DNA methylation) or alter the structure of chromatin by altering scaffolding proteins such as histones, and these changes can be maintained through many cell divisions. It has been shown that epigenetics plays an important role in regulating a wide variety of genes, including the genes involved in the inflammatory immune response [55]. These epigenetic modifications may help to explain the patterns of inheritance seen in asthma and how they interact with environmental factors [56]*.*

In addition to the direct airway damage caused by pollutants, there is a mechanism of epigenetic change in the lung and, in general, all environmental exposures from *in utero* exposure to adult life may lead to epigenetic changes and the development of various disorders [57]. Benzene and PM exposure has been associated with altered DNA methylation. An *in vivo* study of umbilical cord blood DNA carried out at the Columbia Center for Children’s Environmental Health found that the promoter of the *ACSL3* gene was hypermethylated, and that this was related to increased maternal exposure to PAH [58], and the authors of another pediatric study found a significant association between indoor exposure to NO_2_ and severe asthma among children showing a high level of beta-2 adrenergic receptor methylation [59]. Moreover, the level of DNA methylation varies significantly with different types of air pollution. Rossnerova *et al.* concluded that asthmatic children have different methylation patterns, with higher gene expression being found in those living in more polluted areas [60]. Furthermore, a study of 940 children found that short-term PM exposure was associated with less inducible nitric oxide synthase (iNOS) methylation [61].

An increase in histone acetylation has also been observed. One *in vitro* study has found that the exposure of pulmonary tree epithelial cells to PM_10_ involves a global increase in histone H4 acetylation that cause an increase in the production of pro-inflammatory mediators [62]. Furthermore, Cao *et al.* have shown that exposure to DEP increases the expression of COX-2, which causes alterations in chromatin via the mechanism of histone H4 acetylation [63]. Exposure to DEP can also alter microRNA expression in human epithelial cells at the air-liquid interface [64,65]*.*

A more recent study of 20 two-year-old Japanese children found that those living close to major highways, who were more exposed to polychlorinated biphenyle, had higher blood levels of interleukin(IL)-22 mRNA than those who lived further away, and that this tendency was more pronounced in subjects showing IgE food allergy [66].

All of these data support the role of epigenetics in the lung damage induced by air pollution, although these studies were not all appropriately controlled and adequately powered, asthma was defined in different ways and the effects associated with allergic or non-allergic asthma were not differentiated. This highlights that further researches are needed to clarify how and to what extent epigenetics is involved in different populations. Epigenome-wide association studies hold promise for the detection of new regulatory mechanisms that may be susceptible to modification by environmental factors affecting susceptibility to disease [67]. Appropriate study design, a detailed a priori analysis plan and validation of results are essential to minimize the danger of false positive results and contribute to a unified approach.

### Air pollution and acute respiratory infections

Laboratory studies have found that air pollution is associated with severe respiratory tract infection. Spannhake *et al.* demonstrated synergism between NO and rhinovirus infection in human basal and bronchial epithelial cells [68], and it has been shown that concentrated ambient particles increase respiratory syncytial virus replication in mice [69], reduce the capacity of macrophages to phagocytise the virus [70], and reduce *Streptococcus pneumoniae* clearance from the lungs of mice [71]. It is also possible that fossil fuel-derived PM increases the vulnerability to bacterial pneumonia, although it is not clear whether the low PM concentrations common to high-income urban environments have a significant effect [72].

Considering overall the available data, it seems that air pollutants increase the susceptibility to and severity of respiratory infections in the whole pediatric population. Further studies are needed to clarify the advantages that could be obtained with available vaccines (i.e., those against *S. pneumoniae*) and with future vaccines (i.e., those against respiratory syncytial virus) in relation to air pollutants concentrations.

## Conclusions

Air pollution has many negative effects on pediatric health and is recognised as a serious health hazard. However, although there seems to be an association with an increased risk of asthma exacerbations and acute respiratory infections, further studies are needed in order to clarify the specific mechanism of action of different air pollutants, identify genetic polymorphisms that modify airway responses to pollution, and investigate the effectiveness of new preventive and/or therapeutic approaches for subjects with low antioxidant enzyme levels. Moreover, as that epigenetic changes are inheritable during cell division and may be transmitted to subsequent generations, it is very important to clarify the role of epigenetics in the relationship between air pollution and lung disease in asthmatic and healthy children.

## Abbreviations

CAP: Community-acquired pneumonia; DAMPs: Damage-associated molecular pattern molecules; DEP: Diesel exhaust particles; FEV1: Forced expiratory volume in one second; GST: Glutathione-S-transferase; IL: Interleukin; iNOS: Inducible nitric oxide synthase; LPS: Lipopolysaccharide; NO2: Nitrogen dioxide; PAH: Polycycic aromatic hydrocarbons; PAMPs: Pathogen-associated molecular patterns; PM: Particulate matter; PM10: Particulate matter with a diameter of ≤10 mm; ROS: Reactive oxygen secies; TLR: Toll-like receptor; TNF: Tumor necrosis factor; T reg: Regulatory T cells.

## Competing interests

The authors declare that they have no competing interests.

## Authors’ contributions

SE designed the review and drafted the manuscript; RT, ML, VP, EN, SC and MFP performed the literature search, participated in analysing and interpreting the data as well as participated in drafting the manuscript. All of the authors read and approved the final version of the manuscript.

## Pre-publication history

The pre-publication history for this paper can be accessed here:

http://www.biomedcentral.com/1471-2466/14/31/prepub
